# Usnic acid, as a biotic factor, changes the ploidy level in mosses

**DOI:** 10.1002/ece3.3908

**Published:** 2018-02-08

**Authors:** Michal Goga, Dajana Ručová, Vladislav Kolarčik, Marko Sabovljević, Martin Bačkor, Ingeborg Lang

**Affiliations:** ^1^ Core Facility Cell Imaging and Ultrastructure Research University of Vienna Vienna Austria; ^2^ Department of Botany Faculty of Science Institute of Biology and Ecology Pavol Jozef Šafárik University Košice Slovakia; ^3^ Faculty of Biology Institute of Botany and Botanical Garden University of Belgrade Belgrade Serbia

**Keywords:** endopolyploidy, flow cytometry, lichens, mosses, usnic acid

## Abstract

Lichens and mosses often share the same environmental conditions where they compete for substrate and other essential factors. Lichens use secondary metabolites as allelochemicals to repel surrounding plants and potential rivals. In mosses, endoreduplication leads to the occurrence of various ploidy levels in the same individual and has been suggested as an adaptation to abiotic stresses. Here, we show that also biotic factors such as usnic acid, an allelochemical produced by lichens, directly influenced the level of ploidy in mosses. Application of usnic acid changed the nuclei proportion and significantly enhanced the endoreduplication index in two moss species, *Physcomitrella patens* and *Pohlia drummondii*. These investigations add a new aspect on secondary metabolites of lichens which count as biotic factors and affect ploidy levels in mosses.

## 1 INTRODUCTION

Bryophytes (i.e., mosses, liverworts, and hornworts) represent the first green plants which colonized land millions of years ago (Nickrent, Parkinson, Palmer, & Duff, 2000; Shaw, Szövényi, & Shaw, 2011). They developed various adaptations to survive in such a harsh environment such as alternation of gametophytic (haploid) and sporophytic (diploid) generations, elaborate gametophytes or specialized gametangia (Renzaglia & Garbary, 2001). A unique property of bryophytes is their rather small genome size (Goffinet & Shaw, 2009; Vanderpoorten & Goffinet, 2009) as well as their adaptation to various habitats during phylogeny (Bainard & Newmaster, 2010). In fact, they colonize all habitat types except salt water. Apart from facing unfavorable abiotic, environmental conditions, bryophytes also faced biotic stress factors and developed relationships with other organisms to obtain necessary resources.

Mosses and lichens often share similar habitats, where they compete for substrate as well as water, nutrients, and light (Lawrey, 1977; Macías, Molinillo, Varela, & Galindo, 2007). Lichens segregate secondary metabolites to the substrate to disadvantage their competitors. These secondary metabolites represent allelochemicals, which negatively influence vascular plants, mosses, or even lichens nearby (Armstrong & Welch, 2007; Molisch, 1938). As mosses and lichens have a long evolutionary history of allelopathic relations, they achieved survival strategies for cohabitation.

Endopolyploidy is defined as the existence of various ploidy levels in the same individual created by endoreduplication. This endoreduplication is a prerequisite of endomitosis and appears when DNA replication is not followed by mitosis. In general, nuclei with one chromosome set (1C) are monoploid, with two (2C) are diploid and those with three or more chromosome sets are defined as polyploid (Darlington, 1956). For many flowering plants, the ploidy levels of nuclei can vary in different cells, organs, or even tissues (Barow & Meister, 2003; D′Amato, 1964). First insights into endopolyploidy in bryophytes were given by Bainard and Newmaster (2010) who documented its presence in a dozen moss species. Interestingly, endopolyploidy is absent in liverworts and peat‐mosses (*Sphagnum* spp.).

In vascular plants, abiotic factors such as temperature (Engelen‐Eigles, Jones, & Phillips, 2000; Jovtchev, Barow, Meister, & Schubert, 2007), light (Kinoshita, Sanbe, & Yokomura, 2008), drought (Setter & Flannigan, 2001), or salinity (Ceccarelli, Santantonio, Marmottini, Amzallag, & Cionini, 2006) influence the level of endopolyploidy thereby causing effects on growth development as well as stress response (Inzé & De Veylder, 2006). Biotic factors as symbiotic and parasitic relationships also influence endoreduplication in plants (Callow, 1975; Lingua, D'Agostino, Fusconi, & Berta, 2001) fungi, bacteria, and roundworms can all have an impact on the endopolyploidy patterns. For bryophytes, this information is still rare; in particular, the effects of biotic triggers on the ploidy levels are missing. The moss *Physcomitrella patens* is a model organism in plant biology (Cove & Knight, 1993; Reski, 1999). Goga, Antreich, Bačkor, Weckwerth, and Lang (2017) showed effects of lichen secondary metabolite on its development and growth. *Pohlia drummondii* grows usually on heavy metal rich substrates and shares this habitat with lichen species of the genus *Cladonia* (Bačkor, 2011). *Cladonia* is documented to be rich in usnic acid (among other secondary metabolites). The aim of the study was to test lichen compound usnic acid as a biotic factor, which affects the ploidy level in the above moss species.

## 2 MATERIAL AND METHODS

### 2.1 Moss material and culture conditions

Moss plantlets of two species, *P. patens* (Hedw.) Bruch & Schimp. and *P. drummondii* (Mül. Hal.) A. L. Andrews were cultivated under aseptic condition on solid medium. The cultivation medium contained 200 mg/L NH_4_NO_3_, 100 ml/L MgSO_4_.7H_2_O, 400 mg/L KH_2_PO_4_, and 100 mg/L CaCl_2_.2H_2_O, and was solidified with 0.8% agar (VWR, Prolab) at a pH of 5.8 according to Gang et al. (2003). The standard conditions in the culture room were as follows: temperature 22 ± 2°C, 40% relative humidity, 16/8 (day/night) photoperiod, and 83.18 μmol m^−2^ s^−1^ of PAR (photosynthetically active radiation).

### 2.2 Allelopathic assay

Preparation of moss material was carried out according to Goga et al. (2017). In brief, gametophores of *P. patens* and *P. drummondii* were collected from the Petri dish after 5 weeks on control medium and transferred to plastic tubes with deionized water (3 ml/3 gametophores). Subsequently, the moss material was homogenized by a tissue grinder (OMNI TH Homogenizer with Omni Tips^™^). This procedure was carried out for *P. patens* and for *P. drummondii* separately. The homogenous suspension was further used in our allelopathic assay.

Sterilized glass fiber disks (Whatman CF/C filters, glass fiber disks, 25 mm in diameter) were placed on the surface of solid control medium. Usnic acid (UA, Aldrich Company 329967 5C) was dissolved in acetone, and stock solutions of different concentrations were prepared for the treatments (control, 0.01 mg of UA/disk, 0.1 mg of UA/disk). 50 μl of UA, corresponding to the respective treatments, was applied on the surface of each disk by an automatic pipette. Petri dishes with treated glass fiber disks were opened in a laminar flow cabinet for 1 hr to allow the acetone to evaporate. Finally, 40 μl of homogenized moss suspension was applied on each glass fiber disk. Nutrients are able to pass through disk pores from the medium to the plantlets (Bačkor, Klemová, Bačkorová, & Ivanova, 2010). Mosses were cultivated for 5 weeks; each treatment was repeated at least ten times.

### 2.3 Growth area rate

For growth rate analysis, each fiber disk was photographed after 5 weeks and the area occupied by plant material was measured. Images were taken with a camera (Nikon D700, objective Nikon AF‐S 50 mm f/1, 8G). The area on the fiber that was occupied by protonemata and gametophores was quantified using the GSA Image Analysis software (GSA, Rostock).

### 2.4 Flow cytometry analysis of endopolyploidy level

Samples for endopolyploidy analysis were prepared from the whole available plant material grown on respective disks. To isolate cell nuclei, plant material was placed in a Petri dish and chopped in 1 ml of general purpose buffer using razor blades (Loureiro, Rodriguez, Doležel, and Santos (2007); buffer composition: 0.5 mmol/L spermine. 4HCl, 30 mmol/L sodium citrate, 20 mmol/L MOPS, 80 mmol/L KCl, 20 mmol/L NaCl, and 0.5% [v/v] Triton X‐100, pH 7.0). The suspension was then filtered through a 42 μm nylon mesh filter. Nuclei were then treated with 30 μg RNAase and 2 μl mercaptoethanol, and the DNA was stained with 30 μg propidium iodide.

Nuclear ploidy level was determined in a flow cytometer CyFlow ML (Partec Gmbh, Münster, Germany) situated at the Institute of Biological and Ecological Sciences, P. J. Šafárik University in Košice (Slovakia). This laser flow cytometer is equipped with a 532 nm argon‐ion laser. The data histograms were displayed on a logarithmic scale (*x*‐axis) and analyzed with FloMax 2.7 (Partec Gmbh) or FlowJo 10.1 (FlowJo LLC, Ashland, USA) software. The pattern of endopolyploidy was presented as (1) number of peaks appearing in flow cytometry (FCM) histograms, which indicate the presence of nuclei with different ploidy levels in each measured sample and by (2) endoreduplication index (EI) representing the degree of endopolyploidy. EI was calculated according to Barow and Meister (2003) but modified as indicated below because the moss gametophyte represents a life phase with reduced chromosome complement:

$$\text{EI}=\frac{\left(0\times n_{1C}+1\times n_{2C}+2\times n_{4C}+3\times n_{8C}\ldots \right)}{\left(n_{1C}+n_{2C}+n_{4C}+n_{8C}\ldots \right)}$$

where *n*_1C_ + *n*_2C_ + *n*_4C_ + *n*_8C_ … represent the numbers of nuclei with the corresponding ploidy level (1C, 2C, 4C, 8C…). Plant samples with EI < 0.1 are not considered endopolyploid (Barow & Meister, 2003).

### 2.5 Statistics

Statistical tests of equal means or medians, ANOVA/Kruskal–Wallis test in case of three testing groups or *t* test/Mann–Whitney test in case of two testing groups (significance level α = .05 was applied), were performed in Past ver. 3.10 software (Hammer, Harper, & Ryan, 2001). Prior to statistical testing, the normality (Shapiro–Wilk test) and homoscedasticity (Levene`s test) of the data were verified. Figures were created using the ggplot2 ver. 2.2.1 package (Wickham, 2009) in R ver. 3.3.2 environment (R Core Team, 2016).

## 3 RESULTS

**Figure 1:**
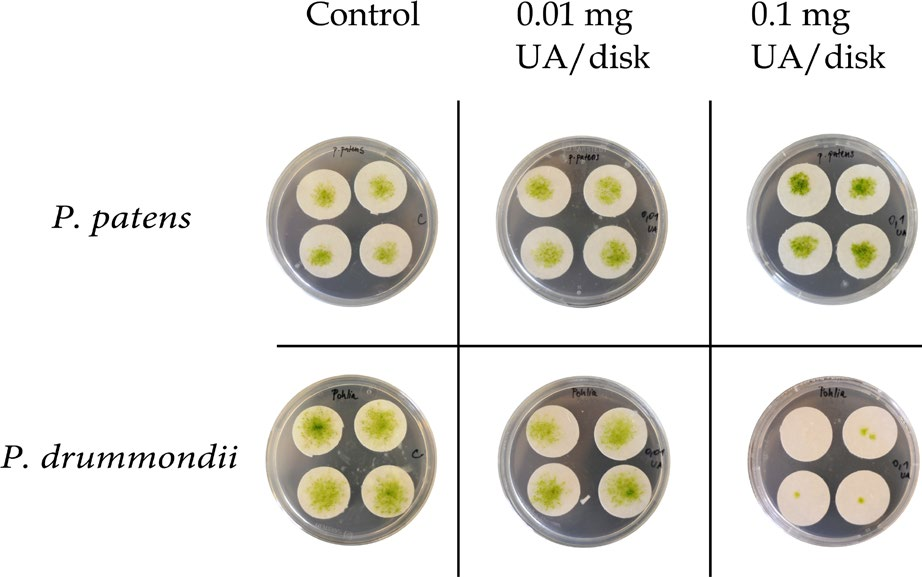
Growth area of *Physcomitrella patens* and *Pohlia drummondii* after 5 weeks of cultivation on fiber disks

**Figure 2:**
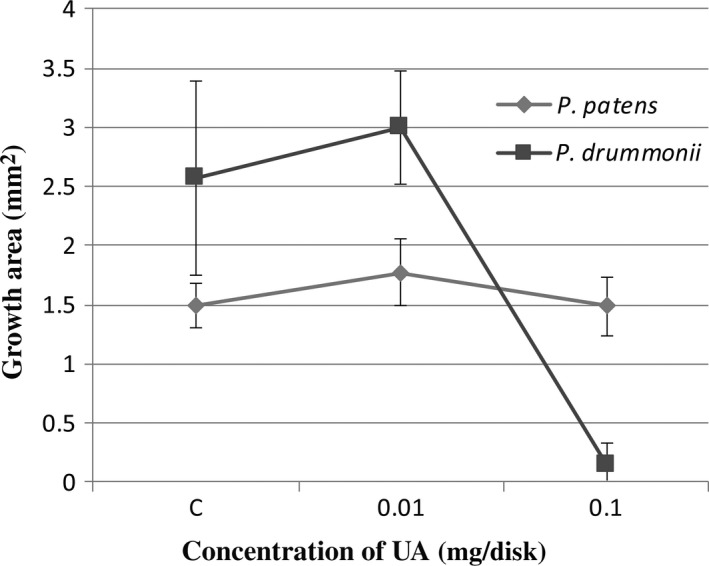
Growth area of *Physcomitrella patens* and *Pohlia drummondii* after 5 weeks. *Physcomitrella patens* is more adapted to exposition of usnic acid (UA) in studied concentration. *Pohlia drummondii* is very sensitive to highest concentration 0.1 mg/disk of UA (*n* = 12)

The growth area experiment was performed on sterile solid media, with homogenized moss material of the two species growing on fiber disks. The disks were treated with different concentrations of usnic acid. After 5 weeks, *P. patens* developed equally fine in control and 0.01 and 0.1 mg UA per disk, and green gametophores were present. In contrast, *P. drummondii* was very affected by the highest concentration of UA (0.1 mg/disk) but grew well in control and the lower UA concentration (Figure 1). Growth area measurements (Figure 2) confirmed that *P. patens* was not affected by UA treatments while *P. drummondii* is much more sensitive to 0.1 mg/disk UA.

**Figure 3:**
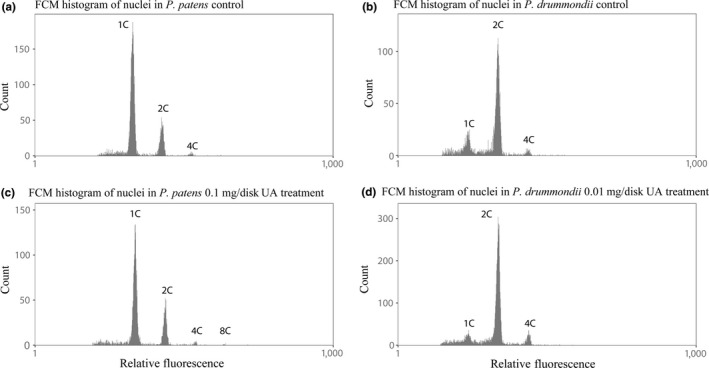
Flow cytometry histograms of both moss species; 1C‐haploid, 2C‐diploid, 4C‐tetraploid, and 8C‐octaploid nuclei. (a) *Physcomitrella patens*, control; (b) *Pohlia drummondii*, control; (c) *P. patens*, 0.1 mg UA/disk; and (d) *P. drummondii*, 0.01 mg UA/disk. UA, usnic acid

**Figure 4:**
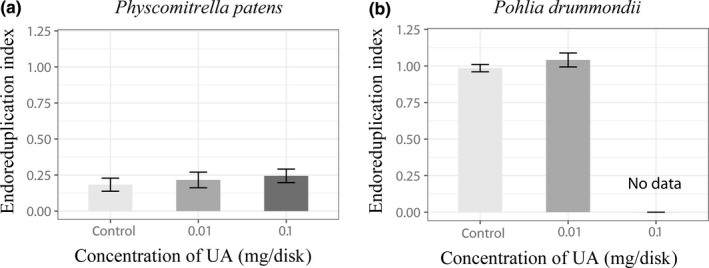
Endoreduplication index in two moss species, *Physcomitrella patens* (a) and *Pohlia drummondii* (b), treated with 0 mg UA (control), 0.01 mg UA, and 0.1 mg UA; error bars represent standard deviation. (No data means, that it was not enough material for measurements). UA, usnic acid

**Figure 5:**
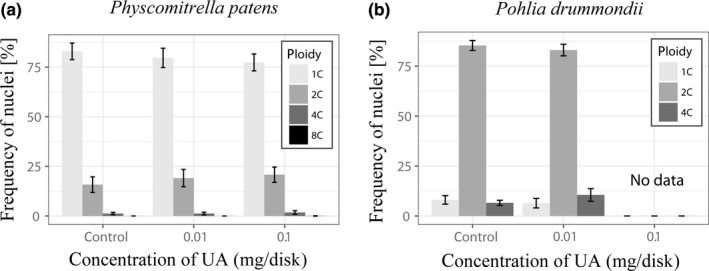
Proportion of nuclei assigned to 1C, 2C, 4C, and 8C ploidy level categories in the moss *Physcomitrella patens* (a) and *Pohlia drummondii* (b) treated with 0 mg UA (control), 0.01 mg UA, and 0.1 mg UA, and error bars represent standard deviation. (No data means that it was not enough material for measurements). UA, usnic acid

Flow cytometry histograms of *P. patens* gametophytes showed 1C, 2C, 4C, and 8C nuclei, but 1C nuclei were most frequent (Figure 3a,c), while in *P. drummondii* (Figure 3b,d), only 1C, 2C, and 4C nuclei present and 2C nuclei were most frequent. Despite relatively low abundance of polyploid cells in *P. patens*, the presence of 4C nuclei was strongly evidenced. The EI in *P. patens* varied between 0.11 and 0.33 (Figure 4a). Therefore, this moss can be considered as an endopolyploid species, in addition to the species tested by Barow and Meister (2003). The proportion of 2C and 4C nuclei increased with UA application, even 8C nuclei were recorded (Figure 5a, Table 1). This is also reflected by the increasing EI which is significantly different between treatments (ANOVA, *p* = .014). Tukey`s pairwise post hoc test revealed significant differences between control and 0.1 UA treatment (*p* = .008241).

**Table 1:** Proportion of nuclei classified according to their DNA ploidy level in two mosses *Physcomitrella patens* and *Pohlia drummondii*. (means that no data were recorded)

	1C	2C	4C	8C
*P. patens*				
Control	82.93 ± 4.19	15.82 ± 3.92	1.25 ± 0.59	—
0.01 UA	79.65 ± 4.87	19.11 ± 4.36	1.27 ± 0.66	—
0.1 UA	77.34 ± 4.26	20.80 ± 4.36	1.47 ± 0.86	0.04 ± 0.09
*P. drummondii*				
Control	8.07 ± 2,15	85.34 ± 2.48	6.59 ± 1.27	—
0.01 UA	6.41 ± 2.37	83.05 ± 2.92	10.54 ± 3.15	—
0.1 UA	—	—	—	—

UA, usnic acid.

In gametophyte of *P. drummondii,* flow cytometry measurements detected three peaks on FCM histograms (Figure 3b,d) which correspond to 1C, 2C, and 4C nuclei. The data suggest that *P. drummondii* is a highly endopolyploid species. Application of UA led to an increase in 2C nuclei proportion (Figure 5b, Table 1) and a significant increase in EI (Figure 4b, *t* test, *t* = 2.46, *p* = .039). The 0.1 mg/disk of UA treatment was excluded from statistics because this treatment was mostly lethal and too little material was available for FCM measurements.

In conclusion, the effect of UA on gametophyte growth is accompanied by an increasing number of endopolyploid cells in gametophytes. Endopolyploidy increased with increase in UA concentration (0 < 0.01 < 0.1 mg/disk) in both studied mosses. However, the EI of *P. drummondii* was higher than in *P. patens*.

## 4 DISCUSSION

We have tested UA, the most common secondary metabolite in lichens, as a biotic trigger for endopolyploidy on two moss species *P. patens* and *P. drummondii*. We demonstrated that this lichen compound strongly influenced the level of ploidy in mosses and acted as a new biotic factor to provoke endopolyploidization.

Usnic acid is present in many lichen species especially in the genera *Alectoria, Cladonia*,* Evernia, Lecanora, Ramalina,* as well as *Usnea*. The concentration of UA in *Usnea* species varies between 0.22% and 6.49% of dry weight (Cansaran, Kahya, Yurdakulol, & Atakol, 2006), in *Cladonia fimbriata*, 0.14% of dry weight was detected (Cansaran Duman, Aras, & Atakol, 2008). Already at low concentrations, this bioactive compound showed strong effects and 0.5–1 μg/ml of UA inhibited bacterial growth (Maciazg‐Dorszyńska, Grzegorz, & Guzow‐Krzemińska, 2014) resulting in great interest of pharmaceutical industries. In a natural habitat where lichens and mosses compete for resources, UA is most likely dissolved by rain. The solubility of UA in water is very low (Jin, Rao, Bian, Zeng, & Yang, 2013), but rain is never pure water because it contains various admixtures, resulting in greater availability of UA thereby causing its allelopathic effect on vascular plants (Bialczyk, Latkowska, & Lechowski, 2011) or algae (Lokajová, Bačkorová, & Bačkor, 2014). Preexperiments in *P. patens* showed that very low concentrations of UA (0.001 mg/disk) caused no significant difference to control (Goga, unpublished data). Higher concentrations were lethal for mosses (Goga et al., 2017), and therefore, we focused on the concentrations used here for studied species.

*Physcomitrella patens* was more resistant to the studied doses of UA, while the highest dose (0.1 mg UA/disk) had strong negative effects on growth area of *P. drummondii*. We assume that the reason for this difference comes from biological and ecological features of the two investigated species. *Physcomitrella patens* does not inhabit the sites with lichens and has a very short life span, while *P. drummondii* shares the habitat and resources with lichens and is coevolutionary adapted to react to smaller doses of UA. Thus, *P. drummondii* reacted by endoreduplication which again favors the survival by expressing vigorous and more competitive moss plants. The production of higher doses of UA, however, means that in competition for resources, lichen can overpower mosses.

Endopolyploidy per se is a phenomenon just at the beginning of exploration and its biological significance is still far from being understood (Bainard & Newmaster, 2010). A comprehensive view of the mechanism(s) and its roles in the plant kingdom in general and bryophytes in particular are rare. Thus, the importance of endoreduplication in the plant kingdom is subject to different hypotheses. One of them suggests that endoreduplication provokes changes in the activity of cyclin‐dependent kinases which are responsible for the normal changeover of the cell cycle (Inzé & De Veylder, 2006). Endopolyploidy might therefore be seen as a response in growth and development. Indeed, Goga et al. (2017) showed that lichen metabolites provoked exactly those effects in *P. patens*. Although the molecular mechanism remained unknown, polyploidization in mosses definitely plays a role, as is evident here. Another hypothesis relates endopolyploidy to the chromosome size. According to Schrader and Hughes‐ Schrader (1955) and Leitch, Chase, and Bennett (1998), chromosome size of natural polyploids is smaller than in related diploids. Accordingly, small chromosome size is a preadaptation to the origin of polyploidy (Darlington, 1956). A third hypothesis supports the “nucleotypic theory” and is based on the fact that cell volume and other phenotypic traits are influenced by DNA amounts (Barow & Meister, 2003). These authors suggested for vascular plants that small genome size and endopolyploidy degree are correlated positively. Therefore, one possible function of endopolyploidy is the compensation of nuclear DNA deficiency especially in species with small genomes. This is confirmed by our research in the two moss species as well. Namely, *P. drummondii* with 11 chromosomes has a higher EI than *P. patens* with 27 chromosomes. Furthermore, the cell type and age played a role in the degree of endopolyploidy in *P. patens* as well, and different ploidy levels were expressed in various tissues (Schween, Gorr, Hohe, & Reski, 2003). In addition, endopolyploidy may be a response in the adaptation to various habitats during phylogeny. In mosses, endopolyploidization per se is documented and various abiotic factors can be triggers for its appearance, level, and expression degree. Those abiotic conditions including temperature, light, and drought cause different levels of endopolyploidy in individual bryophytes species (Bainard & Newmaster, 2010). Here, we show that also biotic factors such as lichen secondary compounds affected the endopolyploidization status in mosses and that it differed in the two investigated species.

## 5 CONCLUSION

In this pioneer study, we demonstrated that lichen compounds influenced the level of endopolyploidy in mosses. We confirm the occurrence of endopolyploidization in two additional moss species *P. patens* and *P. drummondii*, supplementary to 46 bryophyte species previously tested by Bainard and Newmaster (2010). Furthermore, UA as a new biotic factor is documented to affect the endoreduplication in *P. patens* and *P. drummondii*. UA is shown to impose allelopathic effects on both mosses suggesting a role in the natural environment as well. In the competition for the common resources, endoreduplication might enhance the chances of the moss by giving vigorous growth and better development. This is, however, only true for physiological concentrations as higher amounts of UA strongly decrease the developmental potential, at least in *P. drummondii*.

## CONFLICT OF INTEREST

None declared.

## AUTHOR CONTRIBUTIONS

MG, DR, and IL—conceived and designed the experiments; VK and DR—analyzed data by flow cytometry; MG, MS, and IL—performed cultivation of mosses; MG and MB—performed growth data analysis; MG, DR, IL, and MS—wrote the manuscript.
